# Accumulation of RIPK1 into mitochondria is requisite for oxidative stress-mediated necroptosis and proliferation in Rat Schwann cells

**DOI:** 10.7150/ijms.69992

**Published:** 2022-10-31

**Authors:** Baoli Wang, Jiayao Fu, Ying Chai, Yuemin Liu, Yanlin Chen, Junhao Yin, Yiping Pu, Changyu Chen, Fang Wang, Zhiyang Liu, Lingyan Zheng, Minjie Chen

**Affiliations:** 1 Department of Oral Surgery, Shanghai Ninth People's Hospital, College of Stomatology, Shanghai Jiao Tong University School of Medicine, Shanghai, China.; 2 National Center for Stomatology, Shanghai, China.; 3 National Clinical Research Center of Oral Disease, Shanghai, China.; 4 Shanghai Key Laboratory of Stomatology & Shanghai Research Institute of Stomatology, Shanghai, China.; 5 Laboratory of oral microbiota and systematic diseases, Shanghai Ninth People's Hospital, College of Stomatology, Shanghai Jiao Tong University School of Medicine, Shanghai, China.; 6 Department of Neurosurgery, Ren Ji Hospital, Shanghai Jiao Tong University School of Medicine, Shanghai, China.

**Keywords:** Oxidative stress, RIPK1, Schwann cells, necroptosis, Reactive Oxygen Species

## Abstract

The injury of Schwann cells is an important pathological feature of peripheral neuropathy. However, the explicit molecular mechanism and blocking method remains to be explored. In this study, we identified an pivotal executor of necroptosis—RIPK1, performed an unique function in response to oxidative stress-induced injury in Rat Schwann cells. We found that after oxidative stress-simulation by H_2_O_2_, RIPK1 was activated independent of genetic up-regulation, but through the post-translational modification, including its protein levels, phosphorylation of Serine 166 and Serine 321 sites and its general ubiquitination levels. Under a confocal microscopy, we found that RIPK1 was significantly accumulated into the mitochondria. And the phosphorylation, ubiquitination levels were also elevated in mitochondrial RIPK1, as indicated by immunoprecipitation. Through the administration of N-Acetyl-L-cysteine (NAC), a ROS inhibitor, we found that the phosphorylation, ubiquitination and mitochondrial location of RIPK1 was significantly suppressed. While administration of Necrostatin-1 (Nec-1) failed to influence the levels of ROS and mitochondrial membrane potential, revealing that RIPK1 served as the down-stream regulators of ROS. Lastly, pharmacological inhibition of RIPK1 by Nec-1 attenuated the levels of necroptosis, increased proliferation, as indicated by Annexin V/PI evaluation, CCK-8 detection, TEM scanning and EdU staining. Our results indicate a previous un-recognized post-translational change of RIPK1 in response to oxidative stress in Schwann cells.

## Introduction

Oral-maxillofacial peripheral neuropathy, such as trigeminal neuralgia (TN), diabetic peripheral neuropathy and others, can be triggered by several external simulations, including high-glucose microenvironment, mechanical trauma and exposure of several chemical reagents^1^, ^2^. Among these inducers, the hyper-production of Reactive Oxidative Stress (ROS) and the transduction of cell death and inflammatory signaling are the common features of these pathological originals^3^-^5^. Compared to the pathological changes in center nerves system peripheral neuropathy, as TN for example, majorly occurred by the injury of trigeminal nerves, leading to the pain and uncomfortable in peripheral nerve system^3^. Peripheral neurocytes are major composed by Schwann cells (SCs), these cells are distinct to the classical astrocytes in centre nervous system (CNS). It has been widely acknowledged that SCs are crucial elements in peripheral nervous system, which play important roles after peripheral nerve injury^6^, ^7^. After trauma, SCs undergo demyelination and rapidly down-regulate myelination-related genes^8^, ^9^. Several biological process, including necroptosis, apoptosis and inflammatory response were also activated at this time point^10^, ^11^. Injured SCs changed the morphology of peripheral neurons, recruited the migration of macrophages and T helper cells into the peripheral neuron-tissues^12^-^14^. These process has been regarded as the critical pathological features of peripheral neuropathy (PN), leading to pain, inflammation and uncomfortable of peripheral neuropathy patients^15^.

As has been described above, the accumulation of ROS is a pivotal process that induce the abnormal response of Schwann cells, which potentially lead to the pain and inflammatory response after injury^16^. Production of ROS in neuron could be blamed by several sources, usually involving Ca2^+^ influx, glutamatergic stimulation and exposure of hydrogen peroxide 17. Usually, ROS are majorly produced by oxidative phosphorylation (OXPHOS) and play a crucial role in maintaining the redox balance^18^. However, excessive production of ROS have long been considered as a driving force for apoptosis, necroptosis and inflammatory signaling, and may contribute to neuropathic pain and inflammatory pain^19^.

The receptor-interacting protein kinase 1 (RIPK1), has long been regarded as a pivotal necroptosis marker when caspase 8 was inactivated^20^, ^21^. Emerging evidences have indicated that RIPK1 served as a promising therapeutic target for the treatment of a wide range of human neurodegenerative, autoimmune and inflammatory diseases^22^, ^23^. Traditionally, the RIPK1 response to the simulation of TNF and thereby mediating the cell death and systemic inflammatory response in cells^24^. Activation of RIPK1 orchestrated the balance and transduction of its down-stream MAPKs signaling, NF-κB signaling and others, thereby performing multiple roles in addition to necroptosis regulations, such as autophagy, apoptosis and inflammatory response^25^, ^26^. These pathways play pivotal roles in the pathogenesis of peripheral neuropathy. In our previous study, we found that the MAPKs signaling and NF-κB signaling were inordinately activated in peripheral neuro- cells after injury in rat models (Also summarized in Results section). On the other side, accumulating evidences highlighted that cellular ROS accumulation might be correlated with RIPK1 in many cell types^27^-^30^. However, whether the RIPK1 is involved in ROS-mediated neuro-cell injury remains to be explored.

In this study, we explored the role of RIPK1 in response to oxidative stress induced injury at rat Schwann cell lines, and identified that the response of RIPK1 majorly occurred at the levels of post-transcriptional modification rather than gene expressions. Oxidative stress-induced ROS accumulation recruited the RIPK1 into the mitochondria, and modulated the phosphorylation and ubiquitination of RIPK1. Pharmacological inhibition of RIPK1 by Nec-1 alleviated the mitochondrial dysfunction and the NF-κB signaling transduction.

## Materials & Methods

### Cell culture

Rat Schwann Cell Line (RSC96 cells) were kindly provided by the Cell Bank of China. Cells were cultured in D-MEM culture medium supplemented with 10% (v/v) fetal bovine serum and 100U/mL penicillin & streptomycin. For treatment of H_2_O_2_, cells were firstly seeded in 6-plate wells at a density of approximately 50% at the second day, then, different dozes of H_2_O_2_ was added into the culture medium and stimulated for indicated times. Unless otherwise noted, the concentrations for H_2_O_2_-induced RSC96 cell injury were selected at 600 μM, and treated for 8 hrs. The ROS inhibitor, N-Acetyl-L-cysteine (NAC, HY-B0215, MedChemExpress), were utilized at the final concentrations of 10μM in this study. For the inhibition of RIPK1, 500 nM Necrostatin-1 (Nec-1, HY-15760, MedChemExpress) were added prior to H_2_O_2_ stimulation.

### Animal models and behavioral analysis

Male Sprague-Dawley (SD) rats weighting 200-230g were used and underwent with chronic constriction injury of the infraorbital nerve (IoN-CCI), a commonly employed animal model to study neuropathic pain in trigeminal nerve. The rats were given free access to water and food in SPF cages. The animals used in this study were approved in accordance with the animal ethics committee of Shanghai Jiaotong University. The establishment of IoN-CCI rats were based on the criteria of Kernisant et al^31^. Briefly, rats were anesthetized with inhalational isoflurane and an incision was made parallel to the orbit about 1 cm behind the end of the third row of beard line on the near eye side of the face. The infraorbital trigeminal branch was exposed and separated, and a circular ligation was performed with two medical chromium catguts. For control animals, a sham operation was performed without nerve ligation.

The behavior tests are majorly based on the response of mechanical pain thresholds of IoN-CCI rats, through the von Frey test. The test begin on the day before modeling and 1,3,5, 7,10 and 14 after modeling. The collection of infraorbital trigeminal in IoN-CCI and Sham rats were exerted at day 14. Infraorbital trigeminal tissues were digested by 1mg/mL type IV collagenase for 30 minutes, and the cells were collected through Percoll separated layer.

### Mitochondrial isolation

Isolation of mitochondria from cells was performed by a Cell Mitochondria Isolation Kit (C3601, Beyotime, China). The procedure was strictly subjected to the manufacturer's protocol. Of note, the mitochondrial isolation reagent should be kept in 4°C and pre-added with 1mM PMSF (ST506, Beyotime, China). At least 1×10^8^ cells should be collected in each group.

### Immunoprecipitation and immunoblot assays

Collected cells or isolated mitochondria were lysated in ice-cold Immunoprecipitation Lysis Buffer (87788, Thermo Fisher) containing 1% (v/v) Protease and Phosphatase Inhibitor cocktail (78442, Thermo Fisher). For the immunoprecipitation assays, cell lysates or mitochondrial lysates were incubated with anti-RIPK1 at 4°C overnight with gentle shaking. After incubation, Protein A/G beads were further added for a second-round incubation for 1 hr. Then, purified proteins were collected through magnetic beads isolation. Isolated RIPK1 were quantified through micro BCA Protein Assay Kit (23235, Thermo Fisher). Isolated proteins or total protein lysates were separated by SDS-polyacrylamide gel electrophoresis (SDS-PAGE) for immunoblot assays. Polyvinylidene difluoride (PVDF) membranes (03010040001, Millipore) were utilized for protein transfer, incubation and filming. Following antibodies were utilized in this study: RIPK1 (3493, Cell Signaling Technology), p-RIPK1(Ser 321, 38622, Cell Signaling Technology), p-RIPK1(Ser 166, 53286, Cell Signaling Technology), COX4A (38563, Cell Signaling Technology), β-Actin (4970, Cell Signaling Technology), anti-ubiquitin (3936, Cell Signaling Technology).

### Confocal immunofluorescent analysis

Identification of RIPK1 and its association with mitochondria was observed under a confocal microscope (Leica TCS SP8 STED, Germany). Following fluorescent dye for organelles were utilized in this study: DAPI (D9542, Sigma-Aldrich, US), Phalloidin-FITC (17466-45-4, Sigma-Aldrich, US), MitoTracker Red (M7512, Invitrogen, US). For the procedure of immunofluorescent, cells were seeded in confocal dishes (150680, Thermo-fisher, US) and treated as indicated. The Mitotracker Red was added at 40 min prior to fixation. Then, living cells were fixed and permed for 20 min respectively, and incubated with anti-RIPK1 at 37°C for 4 hrs. Secondary antibodies with the fluorescent of Alexa 647 (A32795, Invitrogen, US) were incubated for another 2 hrs. Lastly, cells were washed with PBS for 3 times and sealed for fluorescent image capturing.

Quantitative analysis of the co-localization of RIPK1 and mitochondria was measured by Image J. Briefly, this analysis quantified the fluorescent strength and transferred into the values in Y axis. The position of the fluorescence was transferred into X axis.

### ROS detection assay

At the end of the treatment schedule, RSC96 cells treated for indication were evaluated for intracellular ROS levels through a High sensitive DCFH-DA assay kit (R252, Dojindo, Japan). Briefly, cells were incubated with 10 μM DCFH-DA in culture medium and incubated at 37°C for 30 min. Living cells were either observed under a fluorescent microscope, or evaluated by a Flow cytometry.

### JC-1 based mitochondrial dysfunction analysis

JC-1 MitoMP Detection Kits (MT09, Dojindo, Japan) was used to evaluate the mitochondrial function in RSC96 cells. Briefly, 2 μM JC-1 working solution was added at 30 min prior to collection. After treatment with JC-1, cells were washed with PBS for twice and then cover with imaging Buffer. Fluorescent strength was evaluated under a microscopy.

### Oxygen Consumption Rate evaluation

The evaluation of Oxygen Consumption Rate (OCR) was performed by an instrument of Seahorse XF 96 analyzer. Cells treated with/without Necrostatin-1 were seeded in a 96-well plate and pre-cultured for 24 hrs. The Seahorse XF cell Mito Stress Test Kit containing Oligomycin, FCCP and Rotenone & Antimycin A was utilized and automatically injected at indicated time points.

### 5-ethynyl-2-deoxyuridine (EdU) staining assay

EdU staining assay was conducted by an EdU proliferation kit (C0075, Beyotime, China). Briefly, treated cells were incubated with dissolved EdU reagent for 2 hrs prior to fixation at the indicated time points. Then, embedded EdU was stained with fluorescent dye and observed either by a flow cytometry, or under a fluorescent microscopy.

### Annexin V/PI analysis

The necroptosis detection was determined by an Annexin V/PI Kit (AD10, Dojindo, Japan). Cells were treated as indicated and digested by trypsin for staining. The staining procedures were strictly in accordance with the manufacturer's protocol.

### Transmission electron microscopy (TEM)

RSC 96 cells were collected in 1.5-ml Eppendorf tubes, spun in a refrigerated centrifuge at 4°C, washed 3 times with ice-cold PBS, and fixed in 2.5% glutaraldehyde in 0.1 M phosphate buffer at pH 7.4. Then, cells were post-fixed with 1% osmium tetroxide, dehydrated in a graded ethanol series and embedded in Embed-812 resin. Ultrathin sections (70 nm) were collected on copper grids and double-stained with uranyl-acetate and lead citrate. Cell ultra-structures were observed on a PHILIPS CM120 transmission electron microscope. Analysis of mitochondrial size was referred to the study of Dominguez et al^7^.

### Statistical analysis

All statistical analyses were performed using GraphPad Prism 6 software. Differences in evaluated parameters between groups were tested by two-tailed independent-sample t-tests. Unless otherwise noted, the evaluated parameters in the groups are shown as the means and standard deviations of the mean (SEM) for each group. *p < 0.05 was considered statistically significant.

## Results

### Post-translational modification, but not gene expression of RIPK1 was induced in oxidative stress-induced RSC96 cell injury

**Figure 1:**
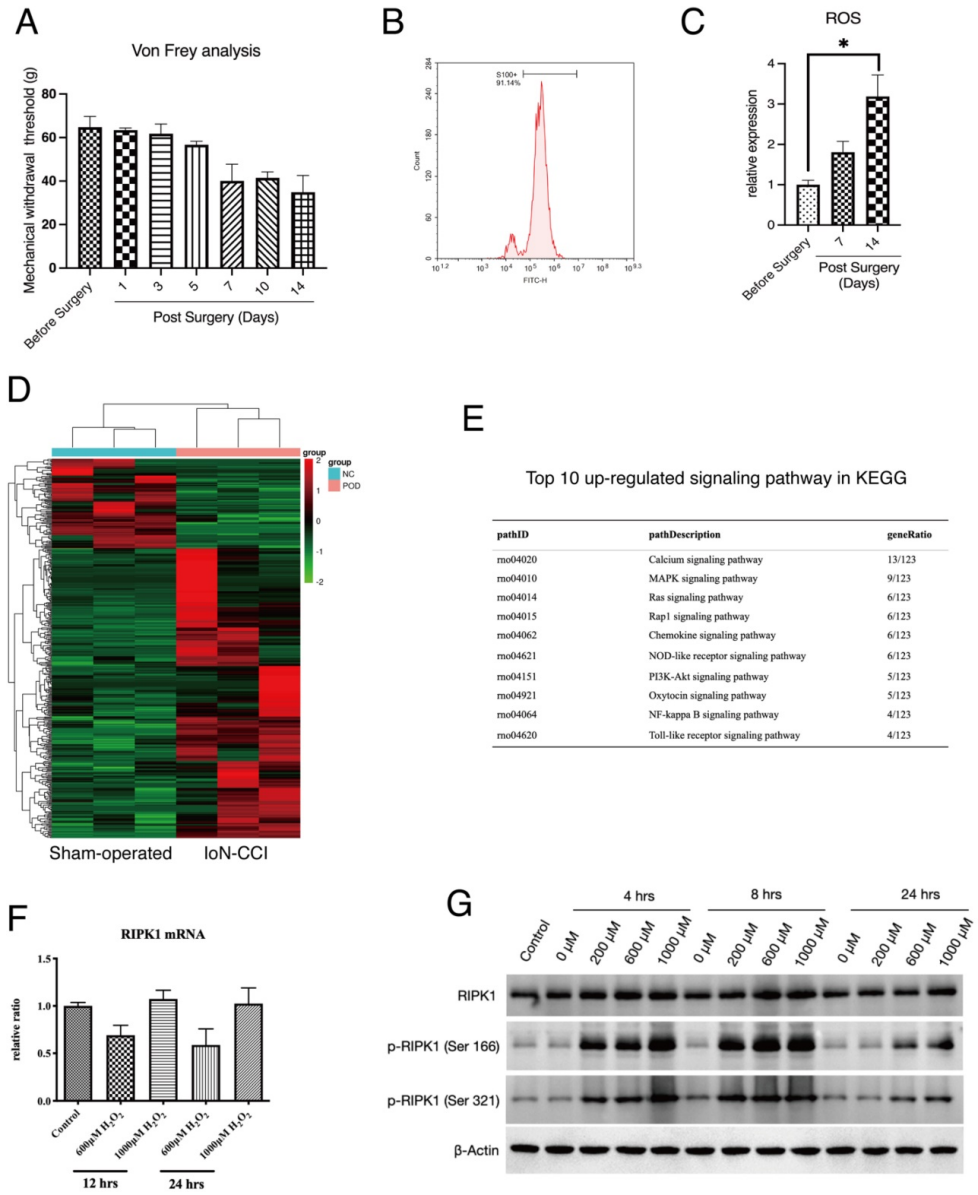
Expression of RIPK1 in oxidative stress-induced RSC96 cells injury. (A) Von Frey measurement of the Mechanical withdrawal threshold of IoN-CCI rat models. n=5. (B) Proportions of S100+ cells (Schwann cells) in extracted neuron tissues. (C) Measurement of ROS concentration in extracted neuron tissues of indicated groups. n=3. (D) Heatmap of high throughput profiling in peripheral neurons of IoN-CCI rat models. n=3. (E) KEGG pathway enrichment of top 10 up-regulated signaling pathway in IoN-CCI rat models. (F) Gene expression of RIPK1 mRNA levels in indicated groups of RSC96 cells. (G) Protein levels of RIPK1, phosphorylated RIPK1 at Serine 166 sites and Serine 321 sites in indicated groups of RSC96 cells.

To detect the potential molecular pathways that was involved in the pathogenesis of PN, we applied a well-recognized IoN-CCI rat model as our *in vivo* investigation target. IoN-CCI rats underwent a mechanical injury will automatically develop a symptom of spontaneous neuropathic pain and accumulation of ROS in neuron tissue, as indicated by previous studies^32^, ^33^. To determine the status of IoN-CCI rats in our experiment, we performed the Von Frey test to quantify the extent of neuropathic pain in rats. The results show that mechanical withdrawn response IoN-CCI rat was significantly changed since Day 5 after surgery (Figure 1A). At Day 14 after surgery, injured neuron tissues were extracted. To identify the component of infraorbital nerves, we applied flow cytometry detection of S100, a hallmark of Schwann cells shows that this cell type occupied the majority of infraorbital nerves (Figure 1B). Lastly, we compared the production of ROS in infraorbital nerves of the indicated groups. The result shows that accumulation of ROS was positively related with the period after IoN-CCI surgery (Figure 1C).

We next extracted the infraorbital nerves of IoN-CCI rats for high-throughput sequencing (Figure 1D). The top-ranked signaling analysis enrichment of sequencing has shown that several down-stream signaling of RIPK1, including NF-κB signaling, MAPK signaling has been activated after peripheral nerve injury (Figure 1E). Therefore, we proposed that whether RIPK1 is involved in this process. To confirm this, we established a previously recommended *in vitro* model — oxidative stress-induced Rat Schwann Cell Line injury, to observe the response of RIPK1 in cells. Several studies proposed that the activation of RIPK1 can be triggered by both gene expression or protein expression^34^, ^35^. However, to our surprise, we found that induction of oxidative stress by H_2_O_2_ failed to facilitate the gene expression of RIPK1 at different time points (Figure 1F). And at a lower concentrations of H_2_O_2_, gene expression of RIPK1 occurred a lower tendency compared to normal conditions (Figure 1F). However, at the protein levels, RIPK1 expression intended to be up-regulated at the time point of 4-8 hrs, or treated with a higher concentrations of H_2_O_2_ (1000μM)(Figure 1G). We therefore interrogated whether the post-translational modification was changed in stress-induced RSC96 cells. As shown in Figure 1G, the classical phosphorylation sites of RIPK1, including Ser 166 site and Ser 321 site, was significantly activated upon H_2_O_2_ simulation, both in time dependent manners and doze dependent manners.

### Oxidative stress triggered the location, phosphorylation and ubiquitination of RIPK1 in mitochondria

**Figure 2:**
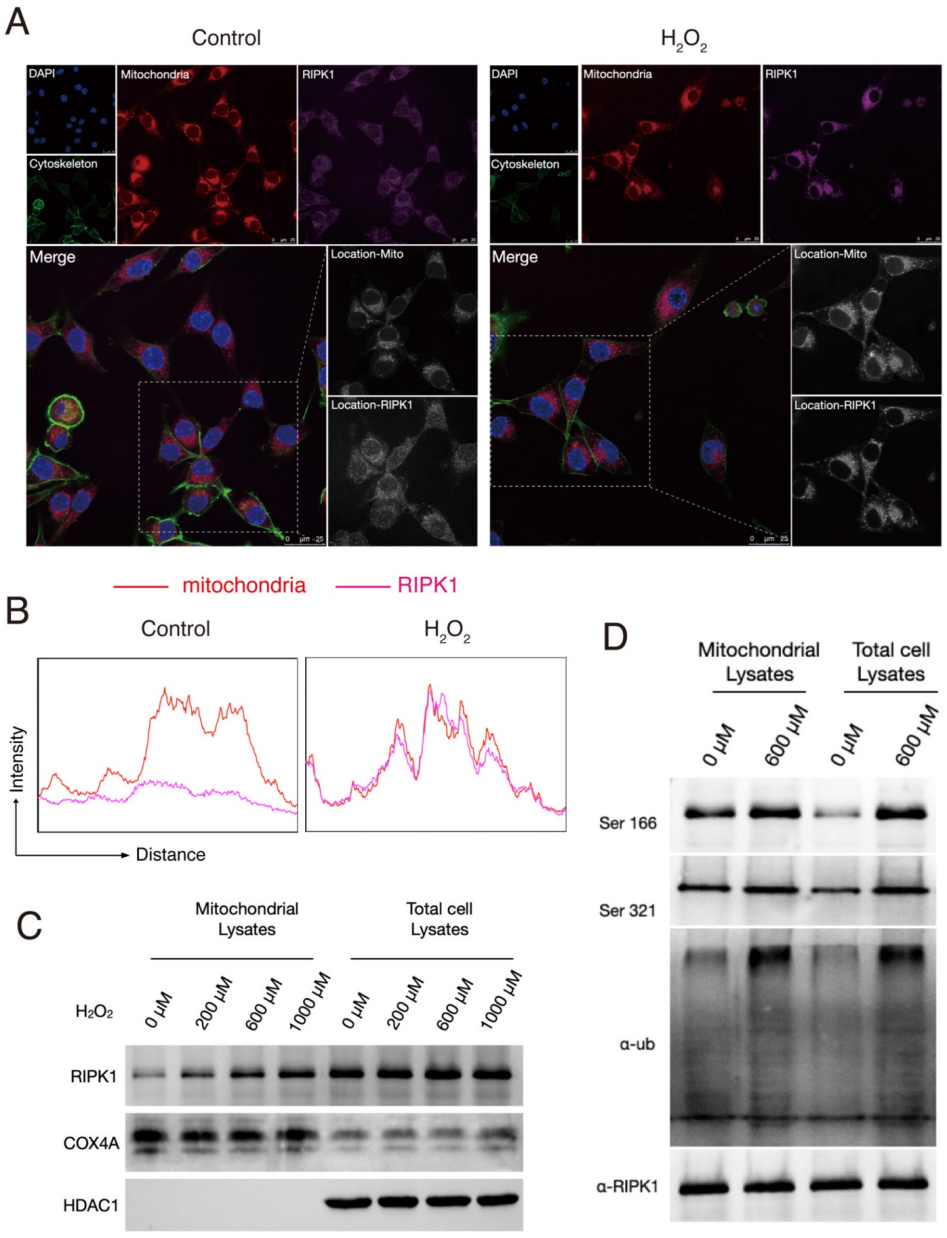
Oxidative stress triggered the location, phosphorylation and ubiquitination of RIPK1 in mitochondria. (A) Representative images of RIPK1 (Violet) and nuclei (Blue), cytoskeleton (Green) and mitochondria (Red) location in indicated groups. The pictures were captured under a confocal microscopy. (B) The quantitative analysis of fluorescent localization of mitochondria (Red) and RIPK1 (Violet) in amplified area of Figure 2A. The X axis indicates the distance of the picture, while the Y axis indicates the density of fluorescent strength. (C) Protein levels of RIPK1 in indicated groups. COX4A was selected for the internal reference protein in mitochondria. (D) Immunoprecipitation analysis of RIPK1 to the phosphorylation and ubiquitination state in mitochondria or total cell lysate of indicated groups.

We next intended to interrogate why and how does the status of RIPK1 changed in response to H_2_O_2_ simulation. To explore this, we firstly stained the RIPK1 with fluorescent antibodies and observed the RIPK1 expression under a confocal microscope. As shown in Figure 1A, at normal conditions, RIPK1 are scattered in cytoplasm of RSC96 cells, while treatment of with H_2_O_2_ accumulated the protein of RIPK1 into several fluorescent plots. As H_2_O_2_ simulation majorly induce the oxidative stress in cells through the excess production of ROS, which induce the dysfunction of mitochondria and other cellular organelles. Therefore, we also co-stained potential organelles including mitochondria, to identify where RIPK1 was located. As expected, we found that location of RIPK1 in H_2_O_2_-treated cells was coincident with the location of mitochondria, compared to unstimulated cells (Figure 2A). To further confirm this findings, we isolated the mitochondria in RSC96 cells treated with/without different dozes of H_2_O_2_. A quantitative analysis of the fluorescent density of RIPK1 and mitochondria shows that under the stimulation of H_2_O_2_, the position of RIPK1 was consistent with mitochondria (Figure 2B). We isolated the mitochondria and purified the protein in different groups, and also, total protein levels were isolated for reference. The results of Western Blots indicate that after H_2_O_2_-simulation, mitochondrial expression of RIPK1 increased significantly compared to the accumulation of RIPK1 in total cell proteins (Figure 2C-D). We also measured the post-translational state of RIPK1 in mitochondria. As has been shown in Figure 2D, upon H_2_O_2_-simulation, the phosphorylation of Ser 166 and Ser 321 site and the ubiquitination levels of RIPK1 were significantly up-regulated, compared to the general levels of RIPK1 in RSC96 cells (Figure 2D). As has been indicated above that after oxidative stress induction, RIPK1 preferred to enter into mitochondria rather than staying in cytoplasm, these results indicate that the entrance of RIPK1 in mitochondria is accompanied by the post-translational modification, including phosphorylation and ubiquitination of RIPK1.

### RIPK1 served as the down-stream signaling of ROS and mitochondria in RSC96 cells

**Figure 3:**
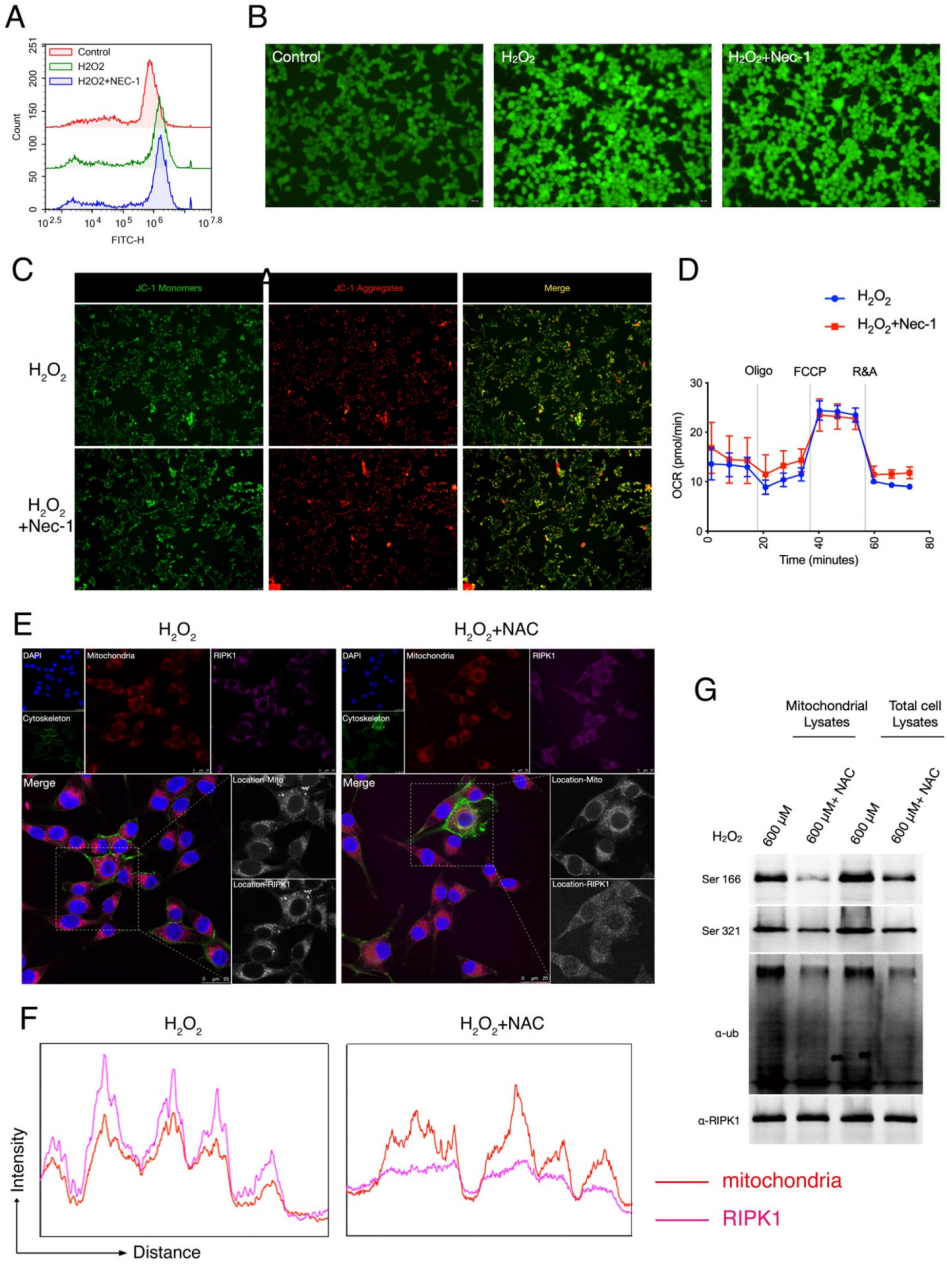
RIPK1 served as the down-stream signaling of ROS in RSC96 cells. (A) Flow cytometry based DCFH-DA analysis in indicated groups. (B) Representative pictures of DCFH-DA staining in RSC96 cells under the fluorescent microscope. The strength of green fluorescence indicate the production of ROS. (C) Representative pictures of JC-1 staining in RSC96 cells under the fluorescent microscope. (D) Real-time Oxygen Consumption Rate in RSC96 cells treated with/without Nec-1. (E) Representative images of RIPK1 (Violet) and nuclei (Blue), cytoskeleton (Green) and mitochondria (Red) location in indicated groups. The pictures were captured under a confocal microscopy. (F) The quantitative analysis of fluorescent localization of mitochondria (Red) and RIPK1 (Violet) in amplified area of Figure 3F. (G) Immunoprecipitation analysis of RIPK1 to the phosphorylation and ubiquitination state in mitochondria or total cell lysate of indicated groups.

Given that after Oxidative stress/H_2_O_2_ simulation, RIPK1 preferred to migrated into mitochondria, and increased its post-translational state in mitochondria. We queried that whether RIPK1 accumulation is responsible for the production of ROS, or whether the migration of RIPK1 could be blamed for the mitochondrial dysfunction, a critical feature after oxidative stress-induced neuron cell injury. To identify this, we treated the pharmacological inhibitor of RIPK1— necrostatin-1 (Nec-1) after H_2_O_2_ treatment. As has been indicated by both flow cytometry and fluorescent microscopy, we found that Nec-1 treatment failed to influence the production of intracellular ROS, through DCFH-DA labelling (Figure 3A-B). We next examined the mitochondrial state after Nec-1 treatment. JC-1 based detection of mitochondrial dysfunction indicated RIPK1 inhibition failed to influence the mitochondrial membrane potential by Nec-1 treatment (Figure 3C). Also, seahorse XF96 based cellular oxygen consumption rate detection indicated that RIPK1 inhibition won't influence the rate of mitochondrial respiration after oxidative stress injury (Figure 3D). These evidences indicate that inhibition of RIPK1 have minor effects to the mitochondrial function itself.

We next look forward to confirm that whether RIPK1 activation served as the down-stream of ROS after H_2_O_2_ simulation. We added the N-Acetylcysteine (NAC), an ROS inhibitor into H_2_O_2_-treated RSC96 cells, and observed the changes of RIPK1 in response to ROS. Under the confocal microscope, we found that after NAC treatment, the migration of RIPK1 into mitochondria was significantly reversed (Figure 3E). This observation was further validated by quantitative analysis (Figure 3F). Furthermore, the ubiquitination and phosphorylation levels of RIPK1 in mitochondria were also significantly descended (Figure 3G). Therefore, we concluded that the changes of RIPK1 served as the down-stream of ROS upon oxidative stress induced injury in RSC96 cells.

### Pharmacological inhibition of RIPK1 restored the balance between necroptosis and proliferation in RSC96 cells

**Figure 4:**
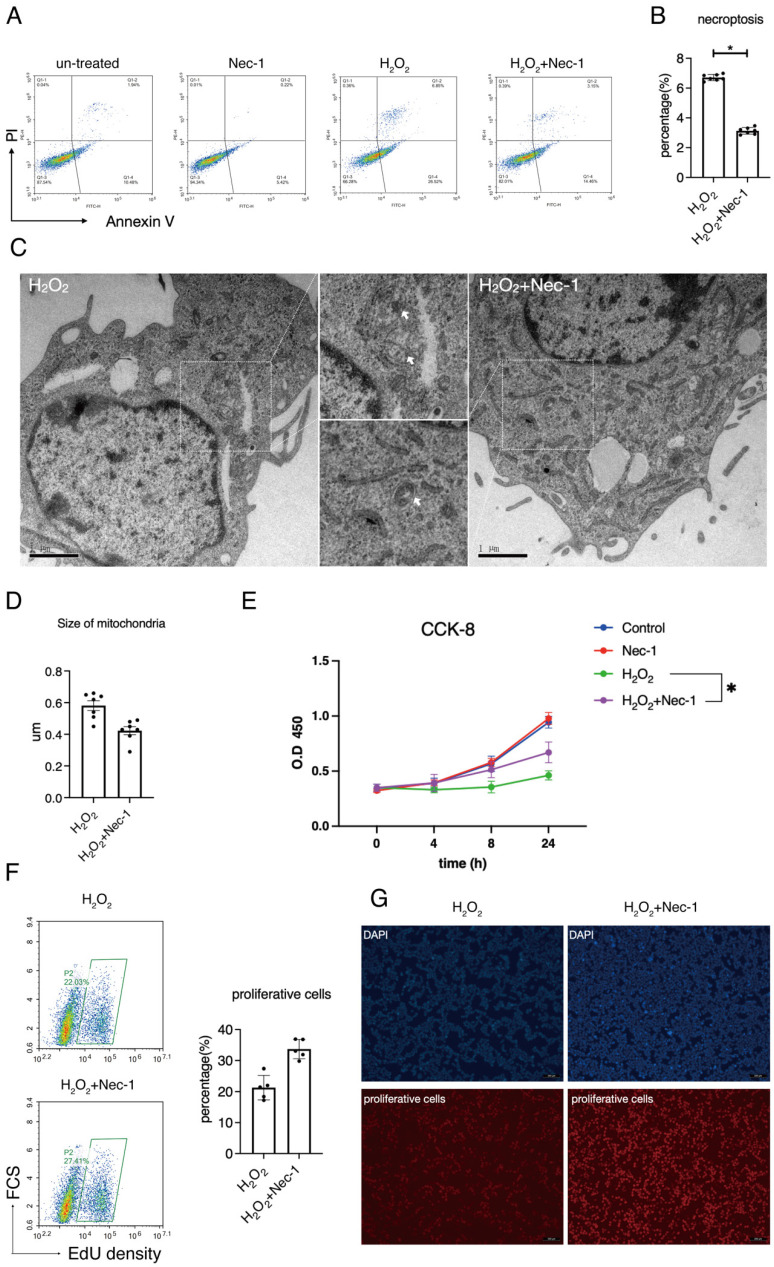
Pharmacological inhibition of RIPK1 restored the balance between necroptosis and proliferation in RSC96 cells. (A) Flow cytometry analysis of Annexin V/PI staining in indicated groups. (B) Statistical analysis of necroptosis rate in indicated groups. n=7. (C) Representative pictures of TEM and observation of organelle swelling in indicated groups. (D) Statistical analysis of mitochondrial size in indicated groups. n=7. (E) CCK-8 cell viability of RSC96 cells in indicated groups. n=3. (F) Flow cytometry analysis and statistical analysis of proliferative cells in indicated groups. n=5. (G) Fluorescent observation of EdU positive cells in indicated groups.

We next aimed at the involvement of RIPK1 to the injury of Schwann cells in response to oxidative stress. Previous studies have established a detailed consensus on the effect of RIPK1 in cells, and considered that RIPK1 might influence several biological features such as necroptosis, apoptosis, proliferation and autophagy, as has been briefly described in Introduction section. Here, we firstly detected the rate of necroptosis (Annexin V^+^/PI^+^) in H_2_O_2_ simulated groups treated with/without Nec-1 through annexin V/PI assays. Flow cytometry detection reveals that inhibition of RIPK1 by Nec-1 significantly down-regulated the rate of necroptosis, (Figure 4A-B). Also, images of transmission electron microscope (TEM) reveals that treatment of Nec-1 attenuated the organelle swelling, a pivotal features of necroptosis in cells (Figure 4C). An analysis of mitochondria in TEM images shows that Nec-1 attenuated the mitochondria swelling due to the size of mitochondria (Figure 4D). Therefore, we concluded that RIPK1 influenced the necroptosis and mitochondria swelling in H_2_O_2_ treated RSC96 cells.

We measured the effect of RIPK1 to the proliferative levels of RSC96 cells. CCK-8 assays show that Nec-1 rescued the cell viability of RSC 96 cells under H_2_O_2_ stimulation (Figure 4E). As indicated by EdU assays, Nec-1 treatment rescued the proliferative ability in cells (Figure 4F). Also, under the microscope, increased numbers of EdU positive cells were found in Nec-1 treated cells (Figure 4G). Collectively, these results indicate that pharmacological inhibition of RIPK1 positively regulate the fate of RSC96 cells, through regulation of both necroptosis and proliferation.

## Discussion

In this research, we identified that the pivotal necroptosis marker— RIPK1, served as a down-stream regulator in response to ROS, and performed its function through the mitochondrial location and post-translational modification in mitochondria, including the transcriptional site of Ser 166, Ser 321 and its general ubiquitination levels. Further studies identified that RIPK1 inhibition orchestrated the balance between necroptosis and proliferation in RSC96 cells. This research highlighted a previously unrecognized molecular mechanism of RIPK1 in response to oxidative stress-induced injury in peripheral neuropathy.

The origin of the oxidative stress can be associated with trauma-, high glucose- and chemical reagent simulation-induced trigeminal neuron discomfort^36^, ^37^. Clinically, Carbamazepine is the first-line drug that can effectively alleviate the clinical symptom. However, the side-effects become apparent with the emergence of drug resistance^38^, ^39^. A substantial proportion of patients have to abandon the drug and choose surgical treatment. The treatment will become extremely troublesome for the patients with recurrent symptoms after surgery, while the recurrent rate ranges from 20% to 50% 5 years after surgery. So, more effective and novel drugs are required to replace the traditional ones. In this perspective, Schwann cells have emerged as an effective therapeutic target to the peripheral neuropathy. Majorly because Schwann cells occupied the major composition of the neuron axons, which delivered the molecular signaling of pain^40^, inflammatory signaling that recruit the macrophages upon peripheral neuropathy development^41^. The exact molecular function of Schwann activation in peripheral neuropathy remains to be explored. However, evidences showed that abnormal demyelination, apoptosis, necroptosis, autophagy of Schwann cells might trigger the development of neuropathy. Among these processes, regulation of RIPK1 to the necroptosis have emerged as a crucial process in nervous diseases, as neuron cells are always resistant to apoptosis^23^. In addition, the structure analysis of RIPK1 shows that there existed an unique hydrophobic pocket in the allosteric regulatory domain, enabling a proper development of highly selective small-molecule inhibitor of its kinase activity^42^. It have been suggested that RIPK1 inhibitors such as Nec-1 are safe to protect the CNS away from neurodegenerative diseases, partly through the inhibition of TNFR1 signaling transduction, but maintained TNFR2-related neuroprotection^43^.

A main discovery in our study is that RIPK1 majorly response to ROS and mitochondria and served as a down-stream regulator of oxidative stress in Schwann cells, these findings are differed from previous reports in other cell types. For example, Wang et al. reported that inhibition of RIPK1/RIPK3 complex suppressed ROS signaling and mitochondrial depolarization in hepatocytes, thereby attenuates liver injury both *in vivo* and *in vitro*^30^. Also, Jia et al. found that RIPK1/RIPK3 activation triggered the necroptosis through JNK1/2-ROS signaling in hepatic stellate cells, and lead to hepatic fibrogenesis with increased extracellular matrix production^28^. On the other hand, some researches also identified similar changes that were consistent with our study. For example, Zhang et al. claimed that ROS promote necroptosis through increase the kinase activity of RIPK1 through autophosphorylation on Ser 161^27^. Some researches even reported there exists a positive feedback loop of ROS-RIPK1 signaling. As Tian et al. found that in an iron overload induced osteoblast necroptosis models, either inhibition of RIPK1 or ROS could attenuate the signaling transduction of others^44^. The conversion of the changes of RIPK1 and ROS in different cell type including Schwann cells hints at a complicated regulatory network between them, which might influence the targeting efficiency in the treatment of peripheral neuropathy.

Also in this study, we found that the ubiquitination and phosphorylation of RIPK1 are activated in mitochondria, and this phenotype seems to be more pronounced upon ROS simulation. It has been well recognized that the post-translational modifications of RIPK1 majorly occurred in complex I, including ubiquitination, deubiquitination and phosphorylation, the abundance of these modification play a crucial role in deciding if the cell will live or die and how it might die^23^, ^45^. For example, highly ubiquitylated RIPK1 in complex I, an transition to an insoluble and ubiquitylated intermediate form that in turn form complex II a/b to induce the necroptosis^45^. While canalization of K63 ubiquitylation site of RIPK1 by E3 ubiquitin ligases cellular inhibitor of apoptosis 1/2 (c-IAP1/2) promoted the recruitment of TAK1, thereby activating IκB kinases and its following NFκB signaling pathway, leading to the production of pro-inflammatory cytokines^46^. Consistent with these changes, the stability of RIPK1 could also been changed. After this study, we intended to further investigate the function of these specific phosphorylation and ubiquitination sites in mitochondria of Schwann cells.

It has been well implicated that the construction of Schwann cells into neuron axis requires high levels of lipid and protein synthesis levels. Therefore, proper functions of mitochondria are critical to the maintenance of Schwann cell myelin and survival. Analysis of Schwann cells in neuropathic peripheral nerves from patients and animal models shows that the mitochondrial function was depolarized and dysfunctional^47^, and Dysfunction of mitochondria is likely to cause peripheral neuropathy^48^. The reason was always because the mitochondria connected the bridge between the external simulation and energy metabolism in Schwann cells. As trauma-induced peripheral neuropathy for example, mechanical stress transferred its signaling into several stress sensor protein, such as TRPV1 and TRPA1^49^. These stress sensor proteins are the classes of Ca^2+^ channel proteins, which subsequently converted the stress to Ca^2+^ influx^50^. And mitochondria, partly performed its function through increase in the Ca^2+^ concentration of the mitochondrial matrix^51^. Then, increased Ca^2+^ concentration elevated the mitochondrial metabolism that accelerated pyruvate dehyodrogenase phosphatase, a-ketoglutarate, and isocitrate into TCA cycle, and thereby, also the production of ROS^47^, ^51^. Ino et al. previously found that the mitochondrial Ca^2+^ uniporter (MCU), plays a key role in myelination^52^. Likely, the binding of MCU and RIPK1 to the Ca^2+^ entry into mitochondria have also been reported^29^. In our study, we found that RIPK1 activation was closely associated with mitochondria in RSC96 cells, providing molecular evidences to the pathogenesis of mitochondrial dysfunction induced peripheral neuropathy.
